# A High-Throughput NanoBiT-Based Serological Assay Detects SARS-CoV-2 Seroconversion

**DOI:** 10.3390/nano11030807

**Published:** 2021-03-22

**Authors:** Taha Azad, Reza Rezaei, Ragunath Singaravelu, Taylor R. Jamieson, Mathieu J. F. Crupi, Abera Surendran, Joanna Poutou, Parisa Taklifi, Juthaporn Cowan, Donald William Cameron, Carolina S. Ilkow

**Affiliations:** 1 Ottawa Hospital Research Institute, Ottawa, ON K1H 8L6, Canada; tazad@ohri.ca (T.A.); rrezaei@ohri.ca (R.R.); rsingaravelu@ohri.ca (R.S.); tjamieson@ohri.ca (T.R.J.); mcrupi@ohri.ca (M.J.F.C.); absurendran@ohri.ca (A.S.); ypoutoupaumier@ohri.ca (J.P.); 2 Department of Biochemistry, Microbiology and Immunology, University of Ottawa, Ottawa, ON K1H 8M5, Canada; 3 Department of Biotechnology, University of Tehran, Tehran 1417614411, Iran; parisa.taklifi@gmail.com; 4 Department of Medicine, Division of Infectious Disease, University of Ottawa at The Ottawa Hospital/Research Institute, Ottawa, ON K1H 8L6, Canada; jcowan@toh.ca (J.C.); BCAMERON@toh.ca (D.W.C.)

**Keywords:** SARS-CoV-2, COVID-19, NanoBiT, serological assay

## Abstract

High-throughput detection strategies for antibodies against SARS-CoV-2 in patients recovering from COVID-19, or in vaccinated individuals, are urgently required during this ongoing pandemic. Serological assays are the most widely used method to measure antibody responses in patients. However, most of the current methods lack the speed, stability, sensitivity, and specificity to be selected as a test for worldwide serosurveys. Here, we demonstrate a novel NanoBiT-based serological assay for fast and sensitive detection of SARS-CoV-2 RBD-specific antibodies in sera of COVID-19 patients. This assay can be done in high-throughput manner at 384 samples per hour and only requires a minimum of 5 μL of serum or 10 ng of antibody. The stability of our NanoBiT reporter in various temperatures (4–42 °C) and pH (4–12) settings suggests the assay will be able to withstand imperfect shipping and handling conditions for worldwide seroepidemiologic surveillance in the post-vaccination period of the pandemic. Our newly developed rapid assay is highly accessible and may facilitate a more cost-effective solution for seroconversion screening as vaccination efforts progress.

## 1. Introduction

The number of patients diagnosed with COVID-19 continues to increase, with more than six hundred thousand daily positive cases and more than ten thousand daily deaths worldwide [1]. The number of vaccine candidates in late-phase clinical trials is increasing, and four vaccines are already approved for public use in several countries [2]. As these global vaccination efforts continue, it is likely that the number of recovered and vaccinated individuals will continue to increase. Sensitive and accurate methods for screening antibody-mediated immunity in vaccinated individuals, as well as in patients recovering from COVID-19, will be needed to better understand the immunity to SARS-CoV-2 in these populations.

Quantification of antibody levels by serological assays is the preferred method for determining post-infection and vaccine-inducible humoral immunity [3]. However, current in-vitro antibody-detection strategies can be time-consuming or lack sensitivity [4]. Thus, developing a novel SARS-CoV-2 antibody detection method with high sensitivity and a fast response time will be critical for studying the evolving immunity to this pathogen in the community. In addition, a SARS-CoV-2 antibody detection assay with reduced overall costs could facilitate greater accessibility of seroconversion detection methods and seroepidemiologic surveillance.

**Figure 1:**
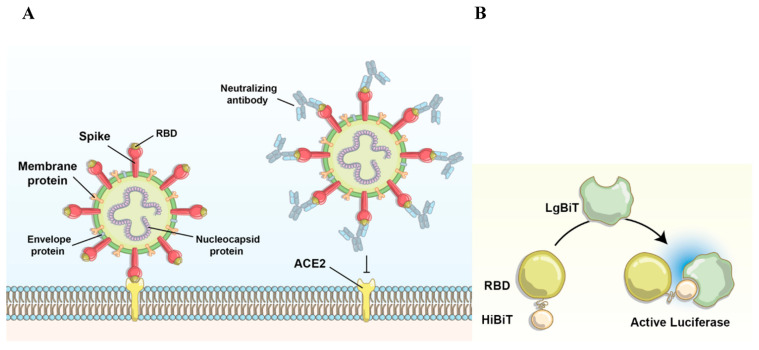
Illustrations of the interactions involving SARS-CoV-2 RBD. (**A**) Interactions between the RBD portion of SARS-CoV-2 and the ACE2 receptor on the host cell can be impaired via neutralizing antibodies. (**B**) Interactions between HiBiT-RBD and LgBiT lead to the generation of bioluminescence signal after substrate digestion.

The receptor-binding domain (RBD) of the SARS-CoV-2 spike glycoprotein interacts with its host receptor, angiotensin-converting enzyme 2 (ACE2), to mediate target cell entry [5,6,7,8] Figure 1A. RBD is the immunodominant target of the humoral response, with the majority of SARS-CoV-2 targeted antibodies and over 90% of neutralizing antibodies in patients targeting RBD. It has also been demonstrated that RBD vaccinated mice elicit an effective neutralizing antibody response [5,6]. The central role of RBD in the humoral response and its poor conservation across coronaviruses makes it an ideal target for the development of serological assays to detect SARS-CoV-2 antibodies. For example, it could be plausible to detect SARS-CoV-2 specific antibodies by using purified RBD fused to a reporter molecule, as long as the signal’s strength and readability is elevated even in blood samples obtained from individuals containing low antibody levels. Moreover, it would be ideal for the assay to be stable at different temperatures and pH to thus remain functional following shipping and handling.

The most widely used bioluminescence reporter systems include firefly luciferase, *Renilla* luciferase, and Nanoluciferase (NanoLuc) [9,10]. Although firefly luciferase is the most commonly used reporter, the NanoLuc system has the highest signal intensity, signal to noise ratio, conformational stability and minimal steric hindrance. This high signal intensity increases the detection range even in the presence of very low amounts of reporter molecules [11]. The split version of the NanoLuc platform, termed NanoLuc Binary Technology (NanoBiT), has a considerably higher stability compared to other bioluminescent reporters [12,13]. The NanoBiT system comprises two segments, a small portion (HiBiT) and a larger portion (LgBiT). The small 11-amino acid HiBiT peptide can be used to tag a target protein with minimal structural interference, to form a bioluminescent enzyme upon binding to LgBiT. There are several reports demonstrating the application of this system to characterize protein–protein interactions [14,15,16] and explore different cellular pathways [17,18]. Such a system has yet to be tested as a highly sensitive, accurate, and reliable method for widespread population serosurveys for SARS-CoV-2. Here, we apply the NanoBit technology linked to the SARS-CoV-2 spike RBD to generate a novel and highly sensitive high-throughput serological assay for detection of seroconversion in patients infected with SARS-CoV-2.

## 2. Experimental Section

### 2.1. Cell Culture

The HEK293T (CRL-3216) cell line was obtained from the American Type Culture Collections (Manassus, VA, USA). Cells were maintained in Dulbecco’s Modified Eagle’s Medium (DMEM) (Thermo Fisher Scientific, Waltham, MA, USA) supplemented with 10% fetal bovine serum (FBS) (Thermo Fisher Scientific, Cat.#SH30396.03) and 1% penicillin-streptomycin (Invitrogen, Carlsbad, CA, USA).

### 2.2. Immunoblotting

Cell lysates were harvested and prepared for SDS-PAGE and immunoblotting, as previously described [6].

### 2.3. Plasmid Design and Transfection

The HiBiT-RBD construct, which includes a secretion signal, in a pcDNA3.1 backbone was ordered from GenScript (Piscataway, NJ, USA). HiBiT-RBD plasmid was transfected into HEK293T cells using PolyJet transfection reagent (SignaGen, Gaithersburg, MD, USA) according to manufacturer protocols. Different concentrations of the HiBiT-RBD plasmid were transfected along with pcDNA3.1 alone as a control to maintain the equal final total plasmid concentration of 1 μg. Supernatants from transfected cells were collected.

### 2.4. Temperature and pH Sensitivity Assay

The supernatants from HiBiT-RBD transfected cells, collected at a volume of 1ml per sample, were incubated overnight at different temperatures (4, 25, 30, 37, 42, 56, or 85 °C), or different pH (range from 2–13), in order to determine its stability. Nanoluciferase assays were performed the following day.

### 2.5. HiBiT-RBD Serological Assay

Patient blood was collected in tubes without anti-coagulant according to The Ottawa Hospital Research Institute protocols and procedures. Samples were collected approximately 3 months after patients tested positive for SARS-CoV-2. After 1 h incubation at room temperature, blood was centrifuged at 5000× *g* for 1 h, and sera was isolated. All serum samples were collected with informed consent from individuals being treated at the Ottawa Hospital General Campus under a protocol approved by the Institutional Ethics Board. The combination of immunoglobulin-binding protein (protein G) and the HiBiT-RBD protein was added to each patient serum sample (5 μL) or the neutralizing antibody solutions (100 ng in 50 μL) in a 96-well plate. Following a 30 min incubation, bound beads were washed three times with PBS to remove unattached HiBiT-RBD. The LgBiT and NanoLuc substrate (furimazine) were added to each sample and then the bioluminescence signals were read using Synergy Microplate Reader (BioTek, Winooski, VT, USA).

## 3. Results and Discussion

### 3.1. Establishment of a Reporter System to Detect Antibody-RBD Interaction

SARS-CoV-2 specific antibodies against RBD typically have a high affinity. We aimed to fuse RBD with the small portion (HiBiT) of the NanoBiT bioluminescence reporter system to detect RBD-binding antibodies. Adding a small tag (11-amino acid) instead of a large reporter protein ensures only a minor change in tertiary structure of RBD, maintaining the antibody binding affinity [19,20]. In this assay, the SARS-CoV-2 Spike RBD antibody binds to the HiBiT-RBD complex, and HiBiT binds to the larger section of the NanoBiT, the LgBiT. The high binding affinity of the two NanoBiT components leads to formation of the fully active enzyme, which digests its substrate rapidly to produce a strong bioluminescence signal (Figure 1A,B).

**Figure 2:**
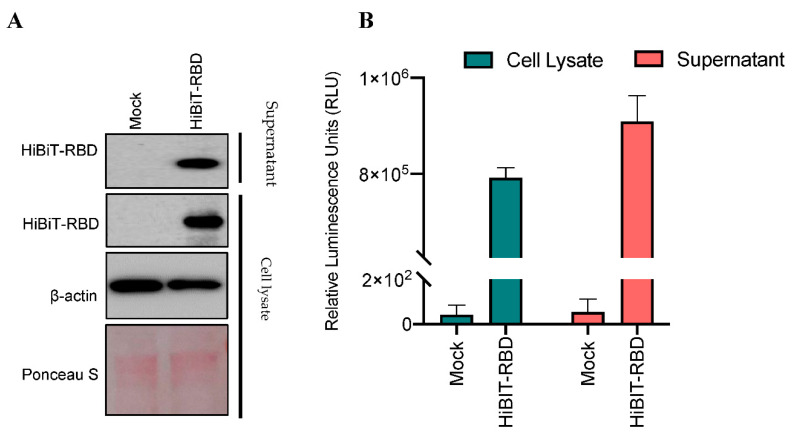
HiBiT-RBD is expressed in both cell lysates and supernatants. (**A**) HEK293T cells were transiently transfected with HiBiT-RBD or pcDNA3.1 empty vector control (1 μg total). Harvested whole cell lysates and supernatants were immunoblotted and probed for RBD. Beta-actin was used as a loading control, and Ponceau staining shows total protein levels. (**B**) Relative luminescence signal with and without LgBiT-substrate treatment of both cell lysates and supernatants.

To optimize HiBiT-RBD protein production, HEK293T cells were transiently transfected with indicated plasmid. Both the cell lysates and the supernatants were evaluated for the amount of active HiBiT-RBD molecules by immunoblotting and luciferase assays (Figure 2). HiBiT-RBD was detected in sufficient quantities in both cell lysates and supernatant fractions.

**Figure 3:**
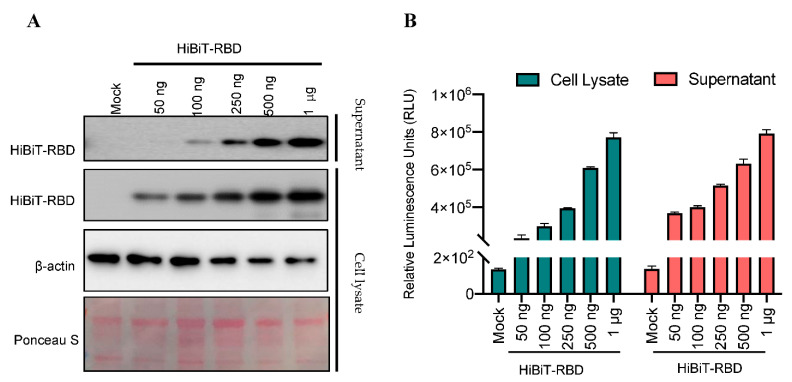
Dose-dependent detection of active HiBiT-RBD in cell lysates and supernatants. (**A**) HEK293T cells were transiently transfected with increasing amounts of HiBiT-RBD (50 ng to 1 μg) or pcDNA3.1 empty vector control (1 μg total). Harvested whole cell lysates and supernatants were immunoblotted and probed for RBD. Beta-actin was used as a loading control, and Ponceau staining shows total protein. (**B**) Relative luminescence signal with LgBiT-substrate treatment of both cell lysates and supernatants.

To validate the production of protein and luminescence signal in a dose-dependent manner, immunoblots and luciferase assays were performed after transfecting increasing amount of HiBiT-RBD-encoding plasmid. As shown in Figure 3, we detected an association between the amount of active protein expression and the concentration of transfected plasmid in the cell lysates and supernatants using two independent readouts, namely immunoblot analysis (Figure 3A) and a luminescence assay (Figure 3B). For ease of protein purification, we used supernatants for subsequent experiments as cell lysates may contain proteins that non-specifically interfere with components of the assay.

### 3.2. High Stability of the HiBiT-RBD System in a Wide Range of Temperatures and pH Levels

**Figure 4:**
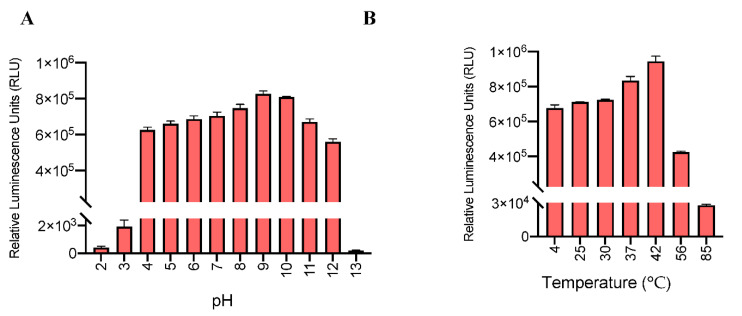
High stability of the HiBiT-RBD reporter system in supernatants at different temperatures and pH conditions. (**A**) Relative luminescence signal with LgBiT-substrate treatment of supernatants from low pH to high pH. (**B**) Relative luminescence signal with LgBiT-substrate treatment of supernatants from 4–85 °C.

To evaluate stability of the HiBiT-RBD reporter, the supernatant pH was set to a range from 2 to 13 (Figure 4A). The reporter actively produced signal even at the lowest pH, but produced the most robust signal between a pH range of 4 to 12. The temperature resistance of the reporter was also assessed by overnight incubation of the supernatants at a variety of temperatures ranging from 4 to 85 °C (Figure 4B). The reporter signal was highly active from 4 to 42 °C, while long exposure to 85 °C led to detectable but significantly decreased signal. The wide dynamic range of HiBiT-RBD activity suggests that this reporter can withstand harsh and long shipping and handling procedures which is necessary for a product that can potentially be used for worldwide serosurveys.

### 3.3. High Accuracy of the HiBiT-RBD Reporter for SARS-CoV-2 Neutralizing Antibodies with Considerable Affinity

**Figure 5:**
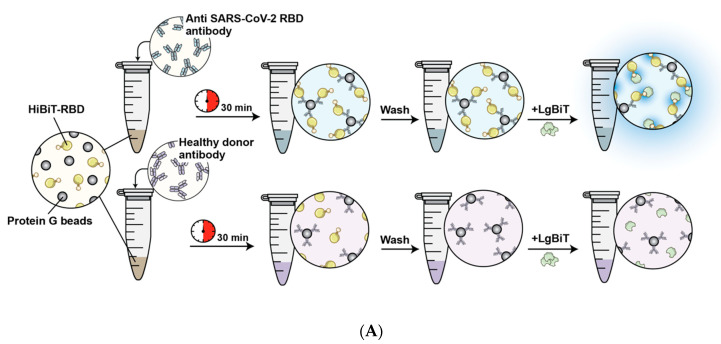

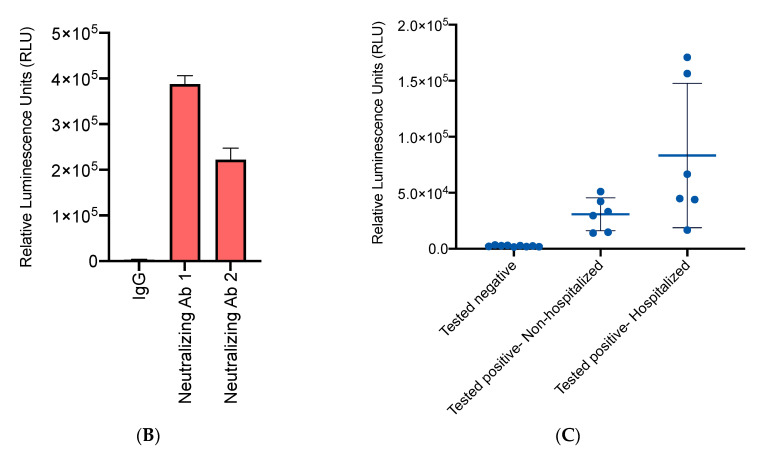
The detection of antibodies using the HiBiT-RBD serological assay. (**A**) Illustration of the NanoBiT serological assay. (**B**) High signal intensity and detection of the SARS-CoV-2 neutralizing antibodies compared to no signal for the control antibody. (**C**) Presence of signal in all positive patient cases and no false-positive results in negative samples. This experiment performed in the 96 well plates which showed the compatibility of the assay for high through put screening.

The HiBiT-RBD reporter was initially validated by testing two different commercial SARS-CoV-2 neutralizing antibodies, SinoBiological 40592 and ActiveMotif 1414, in comparison to control IgG (Figure 5A,B). In this experiment, HiBiT-RBD with protein G was added to 1 μg of commercially available neutralizing antibodies. Following incubation and washing, LgBiT was added and a luciferase assay was performed. The assay enabled detection of both SARS-CoV-2 antibodies with a strong signal, and minimal signal for the control IgG. Also, we tried different quantities of antibodies against RBD and found that 5 ng of antibody in 50 μL assay is the minimum amount of the antibody against RBD and can be detected significantly (Supplementary Figure S1).

### 3.4. High-Throughput NanoBiT Assay Detects Antibody Seroconversion in Recovered COVID-19 Individuals

We next sought the ability of the assay to detect seroconversion in recovered COVID-19 patients. Sera collected from three groups of individuals (i.e., uninfected people, individuals that were infected with SARS-CoV-2 and required hospitalization, or non-hospitalized individuals who contracted SARS-CoV-2) were tested. In this experiment HiBiT-RBD with protein G was added to patient serum. Following incubation at room temperature, beads were washed and LgBiT was added prior to the luciferase assay [Figure 5A,C]. Significant luminescent signal was detected with a range of signal intensities in all serum samples obtained from individuals previously infected with SARS-CoV-2. No false positives were detected among serum samples donated by uninfected individuals that served as negative controls. Interestingly, significantly higher levels of antibodies against RBD were detected in patients requiring hospitalization due to severe illness, compared to non-hospitalized individuals previously infected with SARS-CoV-2. In follow-up studies, we plan to further investigate the wide range of recorded signals and its potential use as an indicator of patient humoral immunity level.

## 4. Conclusions

In summary, we designed and tested a novel serological assay for detection of antibodies against SARS-CoV-2 RBD in recovered COVID-19 patient-derived blood samples. The system is based on the highly sensitive and robust NanoBiT technology and uses HiBIT fused RBD to report on SARS-CoV-2 antibody levels. The stability of the system makes it appropriate for long-term storage in different extreme temperatures, long-distance shipping, and harsh handling conditions. Moreover, the sensitivity of the assay can detect even minimal amounts of antibody in patient samples. The testing procedure takes less than the time necessary for other similar assays or real-time PCR, and the technical requirements are minimal so the test can be performed by most licensed diagnostic laboratory facilities. The full procedure for the assay from beginning to the end is 40 min. We performed this assay in 96 well plate. Measuring luciferase for each plate takes 5 min. So, using our novel NanoBiT-based serological assay, we can easily look at 384 samples per hour. In our study we used 5 μL of serum which will be equal to a drop of blood sample. Also, Supplementary Figure S1 shows that 5 ng of antibody in 50 μL assay is the minimum amount of the antibody against RBD and can be detected significantly. In our assay we found minimal variability among the samples with CV less than 3%, which makes the assay suitable for high throughput screen. This novel NanoBiT serological assay may prove to be a cost-effective method to screen SARS-CoV2 humoral immunity in large populations of recovered or vaccinated individuals. Formal standardization and validation of diagnostic sensitivity and specificity in the clinical context will measure performance characteristics for its seroepidemiological and clinical applications. Our NanobiT based assay in previous reports needed both RBD and ACE2 [21,22,23]. Since ACE2 has very low stability, it makes it difficult for high throughput screening. Our novel detection method presented here works independent of ACE2, involves a simpler methodology, and is more stable.

## Data Availability

Data available on request.

## Supplementary Materials

The following are available online at https://www.mdpi.com/2079-4991/11/3/807/s1, Figure S1: High signal intensity and detection of different amount of the SARS-CoV-2 neutralizing antibodies compared to low signal for the control antibody.
