# Supplementary material for: A High-Throughput NanoBiT-Based Serological Assay Detects SARS-CoV-2 Seroconversion
Source: Nanomaterials (Basel). 2021 Mar 22;11(3):807. doi: 10.3390/nano11030807 (PMC8004173; doi:10.3390/nano11030807)
Supplement: Supplementary file 1 [file nanomaterials-11-00807-s001.pdf]

Supplementary Materials

A High-Throughput NanoBiT-Based Serological Assay Detects SARS-CoV-2 Seroconversion

Taha Azad [1,2,†], Reza Rezaei [1,2,†], Ragunath Singaravelu [1,2,†], Taylor R. Jamieson [1,2], Mathieu J. F. Crupi [1,2,], Abera Surendran [1,2], Joanna Poutou [1,2], Parisa Taklifi [3], Juthaporn Cowan [4], D. William Cameron [4] and Carolina S. Ilkow [1,2,*]

[1] Ottawa Hospital Research Institute, Ottawa, ON K1H 8L6, Canada
[2] Department of Biochemistry, Microbiology and Immunology, University of Ottawa, Ottawa, ON K1H 8M5, Canada
[3] Department of Biotechnology, University of Tehran, Tehran, Iran
[4] Department of Medicine, Division of Infectious Disease, University of Ottawa at The Ottawa Hospital/Research Institute, Ottawa, ON, Canada
* Correspondence: cilkow@ohri.ca; Tel.: +1-613-737-8899 (ext. 75208)
† These authors contributed equally

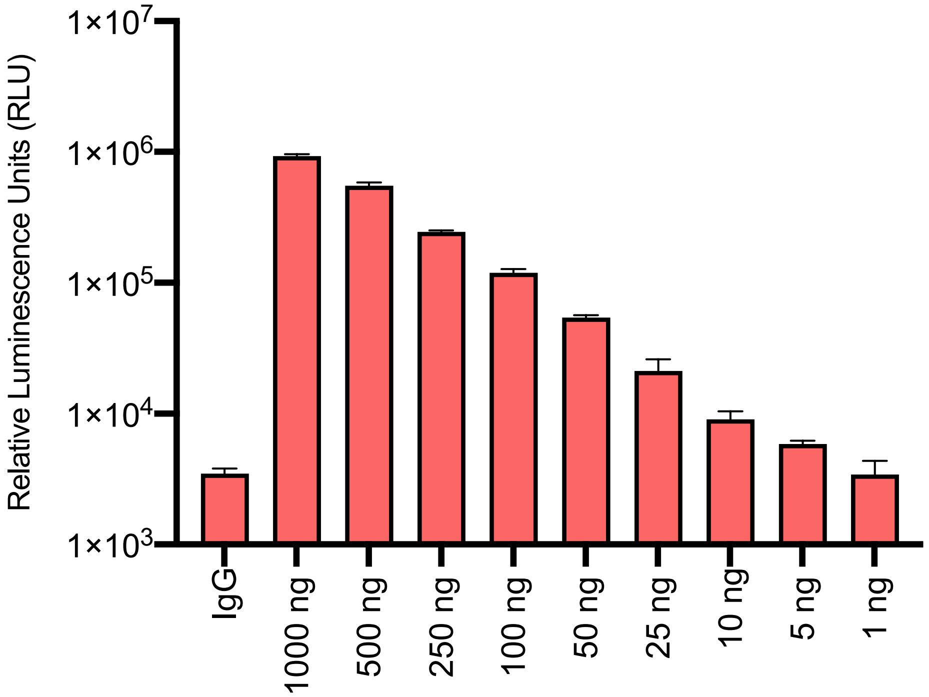

Figure S1. High signal intensity and detection of different amount of the SARS-CoV-2 neutralizing antibodies compared to low signal for the control antibody.
